# The Life of Edward Jenner, M.D. LL.D. F.R.S. &c. With Illustrations of His Doctrines, and Selections from His Correspondence

**Published:** 1838-10

**Authors:** 


### Art. XIII.

*The Life of* Edward Jenner, m.d. ll.d. f.r.s., &c. &c. &c. *With Illustrations of his Doctrines, and Selections from his Correspondence.* By John Baron, m.d. f.r.s., &c. &c. Two Vols.—*London,* 1827-1838. 8vo. pp. 624-471.

The first of these volumes has been some years before the public, and the other anxiously looked for, and quite recently published: they constitute one of the most interesting pieces of medical biography ever composed; and their perusal, whilst it cannot fail to please medical readers of every age, will at the same time be singularly serviceable to every youthful student of our profession, and particularly to all who cherish a hope of enriching medical science and serving the public at large by new discoveries in the science to which they are devoted. The history of Jenner exhibits the life-long efforts of a man of philosophical character and constant habits of observation, catching a glimpse, early in his professional life, of a truth of the highest pathological importance, connected with a discovery of amazing consequence to mankind: we see him keeping this important object ever in view, clearing away the obstacles between him and it, regarding it on every side, and submitting it, with equal industry, candour, and simplicity of mind, to the inspection of every eye; and labouring incessantly to establish it, when fully convinced of its real and incalculable value,—despite of cold disregard and vehement opposition, and misrepresentation, and ridicule, and calumny; but sometimes also with the cheering sympathy of noble minds and the approving judgment of some of the first men of his time,—for the general good of mankind.

Such a spectacle cannot be contemplated too often, or reflected upon too much. Beholding the industry and patience of Jenner, and the partial success which alone crowned his efforts during his lifetime, we learn the useful lesson, that those who aspire to be benefactors of their race must sustain their efforts with a higher hope than that of popular favour; or be exposed to severe discouragement and the chance of desisting from their undertakings whilst they are yet incomplete.

The example set in this respect by Jenner has conferred immortality upon him. Around no tomb is there a purer wreath of fame than that which adorns his memory. Scantily encouraged when living, and almost denied a monument when dead, his life, his labours, his studies, constitute his true glory; for they are those of a mind of a high and pure order, destined to leave its annals recorded in good done to human beings for ever.

Easy and grateful would be the task of following his eloquent and affectionate biographer through the whole career of this great man; to trace not only his public services, but his private character; to paint him in his retirement, as well as in the full observation which he attracted. But the actual state of the public mind, and the anxiety at this time felt by the profession, on the subject which occupied so much of Jenner's thoughts, and which is so inseparably connected with his reputation, demands especial, if not exclusive attention; and to this point, though not without some sacrifice of our own inclinations, we shall at present particularly address ourselves. Amidst many recent demonstrations of public and professional opinion on this subject, very strong manifestations were exhibited at the late meeting of the Provincial Medical and Surgical Association, at Bath, of a general anxiety prevailing in the minds of medical men respecting the reappearance or increased prevalence of small-pox, and the precise degree of protection afforded by vaccination. The proportion of the cases in which it fails to protect, and the causes of this failure, were justly thought of sufficient importance to be made matters of immediate and extensive enquiry by a committee; and it may not be inappropriate to their labours to take a brief and unprejudiced view of some of the steps in the rise and progress of vaccination, from its discovery down to our own day. This we shall be enabled to do, without any apprehension of being wearisome, by following Dr. Baron's lucid order, and by condensing the valuable details with which his work abounds. There will still be left some topics of interest for further consideration, to be hereafter spoken of.

The first intimation received by Jenner of the possible existence of a disease communicated by contact from the cow, and capable of protecting the affected individual from the occurrence of small-pox, was given to him when he was an apprentice with a surgeon at Sodbury: he was doubtless previously acquainted with the popular notion of this, prevalent in that district of Gloucestershire, but it was at this time that it was first impressed on his attention; and from this time he never lost sight of it. Not yet twenty years of age, he contemplated the possibility of delivering mankind from the scourge of one of the most severe and desolating diseases to which human beings were subject: but he was nearly fifty before he published his first work on the Cow-pox. In the interval he had been the pupil of John Hunter, and, on commencing practice in the country, had kept his mind in continual exercise, not only on subjects strictly professional, but in various departments of natural history. He had endeavoured, generally in vain, to stimulate his medical neighbours to take a part in an investigation of which they could not see the importance. They had all seen examples of the alleged protection, but they had also witnessed puzzling exceptions, the explanation of which they wanted patience or genius to unravel. This task he performed himself; and in 1780, after five years of close attention to the subject, and when he was arrived at the age of thirty-one, he was enabled to explain what had perplexed and confused previous enquiry. He at that time stated, to his friend Edward Gardner, his opinion as to the origin of the cow-pox from the heel of the horse; specified the different sorts of disease which attacked the milkers when they handled infected cows, and the particular variety which afforded protection against small-pox, and anxiously and with much emotion communicated his hope that the result of this might eventually be the total extinction of small-pox.

When his mind was much occupied with these views, Jenner made some experiments respecting the nature of swine-pox. He inoculated his eldest son, in 1789, with swine-pox matter; and the result was a disease similar in its progress "to that which arises from the insertion of true small-pox matter, when the disease is very slight. He sickened on the eighth day: a few pustules appeared; they were late and slow in their progress, and small." The subject of this experiment was repeatedly and carefully inoculated for small-pox at subsequent periods, without the slightest effect. Two years afterward, "variolous matter was again inserted by two small incisions through the cutis." Of this the result was as follows. The inoculation was performed April 7th, 1791.

"9th. Evidently inflamed. 10th. An efflorescence, of the size of a shilling, spread round the inferior wound. 11th. The incision assumed a kind of erysipelatous elevation; the efflorescence much increased. 12th. The appearances much advanced. 13th. A vesicle, containing a brownish fluid, and transparent, about the size of a large split-pea, on the superior incision, the inferior about twice as big; the surrounding parts affected with erysipelas. The erysipelas extended to the shoulder, and then pretty quickly went off. The child showed no signs of indisposition the whole time.

"March, 1792. E. Jenner was again inoculated: the matter was taken from a child that caught the disease in the natural way, and had it pretty full. It was inserted fresh from the pustule. The same evening an inflammation appeared round the incision, which, at the end of twenty hours, increased to the diameter of a sixpence; and some fluid had already been collected on the lips of the scratch, which the child had rubbed off." (Vol. i. p. 130.)

This experiment, therefore, Dr. Baron observes, strongly corroborates the opinion, founded on other facts, that the swine-pox, the small-pox, and the cow-pox "had one common origin, and were, in fact, varieties of the same affection."

Several difficulties presented themselves to Jenner in the preliminary part of his enquiry. He soon found that what was commonly *called* cow-pox was not a certain preventive of small-pox. This circumstance was readily explained by ascertaining that cows were subject to various eruptions on their teats, of which the true cow-pox was but one. More perplexity was occasioned by the indubitable fact that even the true cow-pox did not always protect: but of this fact, which seemed at first sight formidable enough to put an end to the whole enquiry, Jenner first divined, and then by observation proved, that the cause was to be sought in the precise period of the disease in the cow at which the virus was communicated to the milker.

At length, on the 14th of May, 1796, the first attempt was made by Jenner to communicate the cow-pox infection by virus taken from the hand of a person casually affected by it, and inserted by artificial inoculation in the arms of another human being.

"Matter was taken from the hand of Sarah Nelmes, who had been infected by her master's cows, and inserted by two superficial incisions into the arms of James Phipps, a healthy boy of about eight years old. He went through the disease apparently in a regular and satisfactory manner; but the most agitating part of the trial still remained to be performed. It was needful to ascertain whether he was secure from the contagion of small-pox. This point, so full of anxiety to Dr. Jenner, was fairly put to issue on the 1st of the following July. Variolous matter, immediately taken from a pustule, was carefully inserted by several incisions, but no disease followed." (Vol. i. p. 137.)

A letter of Jenner's is given, addressed to his friend Gardner, in which he relates this experiment; and he states that, having never before seen the disease except in its casual form, he was astonished at the close resemblance of the pustules, in some of their stages, to the variolous pustules.* Certainly no event so interesting to mankind as this had taken place since the discovery of the practice of inoculation; and of the vaccine inoculation the benefits were to be immeasurably greater.

From this period until 1798, further experiment was prevented by the disappearance of cow-pox from the dairies. In these circumstances, Dr. Jenner made many efforts to generate the disease from the heel of the horse; having adopted the opinion that the *grease* was the origin of small-pox and cow-pox: but there were difficulties in the way of these trials not easily overcome. Although this opinion was not correct, it has since been clearly established that the grease is the form in which the cow-pox affects the horse; and that matter taken from it will produce a pustule resembling the vaccine pustule, and possessing the same protecting power, without passing through the constitution of the cow. Of the latter fact Dr. Jenner was not aware when he published his Inquiry. It appears that there are at least four animals (the horse, the cow, the sheep, and the goat,) affected with a disease communicable to man, and similarly protective from what seems a more malignant form. The dog, the goat, the ass, and the sheep are capable of receiving the vaccine disease by inoculation.

Dr. Jenner's "Inquiry into the Causes and Effects of the Variolæ Vaccinæ," by which his great discovery was first communicated to the public at large, appeared in the form of a thin quarto, of scarcely more than seventy pages, in June, 1798; and was dedicated to his friend the late celebrated Dr. Parry, of Bath. The following views occupy the first few paragraphs:

"The deviation of man from the state in which he was originally placed by nature seems to have proved to him a prolific source of diseases. From the love of splendour, from the indulgences of luxury, and from his fondness for amusement, he has familiarised himself with a great number of animals, which may not originally have been intended for his associates.

"The wolf, disarmed of ferocity, is now pillowed in the lady's lap. The cat, the little tiger of our island, whose natural home is the forest, is equally domesticated and caressed. The cow, the hog, the sheep, and the horse are all, for a variety of purposes, brought under his care and dominion.

"There is a disease to which the horse, from his state of domestication, is frequently subject: the farriers term it *the grease*. It is an inflammation and swelling in the heel, from which issues matter possessing properties of a very peculiar kind, that seems capable of generating a disease in the human body (after it has undergone the modification that I shall presently speak of,) which bears so strong a resemblance to the small-pox that I think it highly probable that it may be the source of that disease." (Vol. i. p. 146.)

* The same remark was made by Soemmering, who had been much accustomed to the minute study of the eruption in small-pox: he was struck with the identity of cow-pox to small-pox in its mildest form; and particularly where there had been but *one* small-pock, a "pearl-like pustule." The very same remark was also made by Ch. L. Hoffman, whom Soemmering calls "a lynx-eyed man," although then eighty years of age. (See vol. ii. p. 216.)

Twenty-three cases were given, illustrative of the progress of the infection; the first sixteen being examples of the casual disease, and the rest the result of inoculation. Among the former is the case of an individual who was infected from the heel of a horse, and afterwards completely resisted the small-pox contagion: among the latter is mentioned that of his second son, Robert Jenner, who "did not receive the infection;" a circumstance rendered important by the circumstance that, at a later period, the said Robert, having been accidentally exposed to the contagion of small-pox, and being of course unprotected, as he had not gone through the cow-pox, was inoculated at once (Jenner having no vaccine virus at the time) for small-pox, as the only security in that emergency against the severity of the natural disease: of this fact the enemies of Jenner, of course, made the most. Dr. Jenner states it as his belief, in the Inquiry, that the facts it brought forward proved "that the cow-pox protects the human constitution from the infection of small-pox;" whilst the opinion that the source of the infection of cow-pox "is a peculiar morbid matter arising from the heel of the horse" was also supported by sufficient evidence, although not proved by actual experiments made under his own eye. At that time he thought that the matter of grease only acquired its protective power after passing through the system of the cow; but subsequent trials proved that this was not a necessary condition.

It is a strange fact in the history of mankind, that the same people who run so hastily in quest of quack medicines, and so readily submit to dangerous methods of counter-irritation, undeterred by the clearest cases of murder; the same people who believe in the astonishing virtue of the billionth part of a grain of camphor, or the curative influence of magnetic manipulations, should, in the case of Dr. Jenner's great discovery, have exhibited such excessive cautiousness that, in the whole course of three months which he passed in London, soon after the publication of his Inquiry, he could not obtain one individual as the subject of the vaccine experiment. He actually quitted London, after three months of patient or impatient waiting, without having vaccinated a single person. Mr. Cline, being supplied with some vaccine virus, did indeed insert it into the hip of a patient who had disease of the hip-joint; but this was done by way of counter-irritation. The patient, however, had the variolæ vaccinæ, and was subsequently inoculated for the small-pox in three places, "which were slightly inflamed on the third day, and then subsided." Mr. Cline became fully aware, it would seem, of the importance of Dr. Jenner's discovery, from the circumstances of this case, although he failed to propagate the vaccine infection from it. As Jenner was at that time unprovided with a supply of vaccine matter, an interval was offered in which the illiberal industriously derided the alleged discovery. Jenner felt this kind of misrepresentation very keenly, and, in reply to Mr. Cline's exhortations that he should remove to town, prophetically foretold the confusion that would, in all probability, arise before the true cow-pox matter was accurately distinguished from the spurious; especially as he foresaw that, even after the disease had been truly gone through, the common process of inoculation would in some cases produce inflammation, vesication, and even pus, in the wounded part, with some constitutional symptoms; circumstances, of which the enemies of science would avail themselves; although, as he observed, the same thing had happened again and again to those who had had the small-pox. (Vol. i. p. 150.)

We may here mention, as interesting facts connected with the early history of vaccination, that, four months after Dr. Jenner left London, he vaccinated the children of his friend Mr. Hicks, of Eastington, the first gentleman who submitted his children to the practice. The first person of rank who allowed her child to be vaccinated was Lady Frances Morton (afterward Lady Ducie). Several ladies, as well as gentlemen not of the medical profession, subsequently lent their personal aid to promote vaccination. Mr. Hicks became an experienced practical vaccinator, and was the first person to practise, in a doubtful case, a second vaccination a few days after the first: he observed the second vaccination making "immense strides to overtake the first;" and this remark became the foundation of the test proposed by Mr. Bryce, of Edinburgh. This was, perhaps, the only improvement not suggested by Jenner himself.

Dr. Jenner, says Dr. Baron, "always considered small-pox and cow-pox as modifications of the same distemper; and that, in employing vaccine lymph, we only make use of means to impregnate the constitution with the disease in its mildest, instead of propagating it in its virulent and contagious form, as is done when small-pox is inoculated." (Vol. i. p. 162.) To the illustration of these views a considerable portion of Dr. Baron's first volume is devoted. With great learning and research he endeavours to show,

> "First, that an eruptive disease, *common* both to man and to the inferior animals, has been known in different ages and in different countries; and that the descriptions given of this eruptive disease by various writers accord so completely with those acknowledged to be characteristic of small-pox, as to render it highly probable that this disease actually existed at a much earlier period than that usually assigned to its origin.
>
> "Secondly, that as there are numberless writers who have described the small-pox in man, so there are others of established name and reputation who have treated of a similar eruptive and pestilential disease as existing, in various countries and in different times, among the inferior animals, but especially among cattle; that to this disease they have unhesitatingly applied the name of Variola, and actually recommended such treatment as experience had proved to be useful when that disease attacks man." (Vol. i. p. 163.)

The numerous passages quoted from writers of various ages in support of these views are not of equal weight; but several of them seem almost decisive, and all deserve attention. As bearing on the appearance of a pestilential and eruptive disease among cattle, closely resembling variola, the account given by Dr. Layard, in the Philosophical Transactions for the year 1780, leaves scarcely a doubt upon the subject. The disease described by Layard was an eruptive fever of the variolous kind, prevalent among horned cattle, and rendered more favorable by inoculation: the epidemic prevailed on the coast of Friseland, North and South Holland, Zealand, and Flanders; but not in the similar coast and climate of the east of England. At other periods it was observed in England and in Picardy, where an account was given of it by Vicq d'Azyr; who agreed with Layard in considering it closely allied to the variolous eruption. Some of the animals had the neck covered with the eruption. Dr. Baron observes, that Layard's experiments by inoculation give an interest and a confirmation to the truth that some of the inferior animals are liable to small-pox in some of its worst forms; and he remarks, that if the disease observed by Dr. Layard had been mild instead of malignant, and if he had attempted to communicate it to man by inoculation, he might have anticipated Jenner's great discovery. It was then of less consequence than it now is to illustrate the connexion between the diseases of man and of the inferior animals, and no attempts were made to ascertain whether the variolæ could be communicated from man to the brute, or from the brute to man. The latter point is established by the discovery of the variolæ vaccinæ; and some experimenters appear to have established the former. Dr. Baron refers to M. Viborg's statements in the Medical and Physical Journal for September, 1802, that he communicated human small-pox to dogs, apes, and swine; and that it had been proved, at the Royal Veterinary College of Berlin, that the cow likewise receives the small-pox by inoculation. (Vol. i. p. 216.)

As Dr. Baron considers the evidence disclosed by the phenomena of Variolæ Vaccinæ strongly corroborative of the historical proofs adduced in support of the above-quoted propositions, and that this evidence is rendered more complete by the history of Variola, as it has been recognized in man, he devotes a chapter of his first volume to the history of Variola and of Variolous Inoculation. It is his opinion, we have seen, that the small-pox was known to and described by authors long before the date to which its origin is usually referred; and even 1500 years before the Christian era. The first recorded case of small-pox, under that peculiar name, is probably, he observes, that of Elfrida, the daughter of Alfred, A.D. 907; and the next in the monkish annals is that of her grandson Baldwin, who died of it in 961; although in the interval it cannot be doubted that many cases occurred, which escaped accurate description. The name of Variola, with its derivatives, seems to have been adopted "by the Latin family of Europe;" and that of pocca, or pock, or little pouch, by the Teutonic or Saxon. In the eleventh century, Avicenna first gave a clear account of small-pox and measles: he distinguished the confluent and distinct small-pox, and represented the measles as different from small-pox, "in a degree at least." Like Rhazes, he observed that "*small-pox may affect the same individual twice.*" Other writers continued to confound the two diseases. In the fifteenth century, Fernelius still included small-pox and measles among pestilences, treating of neither by name. Dr. Mead, says Dr. Baron, promulgated without due enquiry the opinion that small-pox could not affect the same individual twice; contrary to what was evidently the general belief among the early writers: and consequently, from Mead's time until within a few years, the second occurrence of small-pox was always looked upon as an attack of chicken-pox, swine-pox, or some other eruptive disease. The assertion of the friends of vaccination that second attacks did occur was disregarded; but Dr. Baron observes, that more than 130 different writers have recited examples of its occurrence. Louis XV. died of a second attack of small-pox, when sixty-four years of age. The fact of such liability is now universally admitted.

Among the innumerable and almost forgotten attempts to deprive Jenner of the honour of originality, it may be instructive to mention that one was an assertion that Boerhaave had contemplated something analogous to his discovery: but Boerhaave's idea, as clearly set forth in his aphorisms, quoted by Dr. Baron, (*Aphor.* 1388 to 1392,) was merely that of finding some specific preventive of small-pox in the shape of a medicine; and he suggested a combination of mercury and antimony. To such frivolous and vexatious attacks, however, are those exposed whose views are really original, and whose motives are the purest.

In 1717, Lady Mary Wortley Montague caused her son Edward, then a child, to be *inoculated* for the small-pox, at Constantinople, by Mr. Maitland, surgeon to her husband, then British ambassador there. In 1722, her daughter was inoculated, by the same surgeon, in England, "this child being the first known subject of the new practice in civilized Europe." To this accomplished and enterprising woman, therefore, we are indebted for a practice which at least deprived the small-pox of much of its virulence, although it caused its prevalence to be greater than before. For the honour of medicine it should be mentioned that the second child inoculated in England was a child of Dr. Keith. The royal family soon set a courageous example of the same kind; but the practice made way very slowly; particularly as it was soon discovered that cases of small-pox after inoculation were often severe. In eight years after its introduction, 845 persons were inoculated, of whom seventeen, or nearly one in fifty, died. Denunciations of the practice were thundered from the pulpit; and similar ones have been subsequently levelled against vaccination. But Dr. Baron justly observes that many of the clergy countenanced the new practice. We gladly acknowledge the aid given by the same learned body to the diffusion of vaccination. In 1740, the practice of inoculation had nearly fallen into disuse. Subsequent accounts from America and the West Indies, revived its credit, and "in 1746 the Small-pox Hospital in London was founded for the purpose of inoculating the poor, and of keeping the patients distinct from the general population of the city;" the latter regulation being soon unfortunately departed from. In 1752, notwithstanding confident assertions to the contrary, it appears that the deaths from small-pox had actually increased. In 1763, the practice of inoculation was prohibited in Paris, by royal authority, in consequence of the great and fatal increase given by it to the diffusion of small-pox. In Spain the practice was scarcely ever admitted; and there, it is said, and Dr. Baron believes, the small-pox has occasioned less mortality than in any other country. The conclusion which we must arrive at, with Dr. Baron, is that without proper restrictions and careful seclusion of the infected, inoculation does but augment the evils of small-pox by increasing the number who are affected, and severely affected. In 1786, the Empress Catherine invited Baron Dimsdale to Petersburgh, and submitted herself and her son Paul to inoculation: the practice became fashionable; and the small-pox became so prevalent, that Sir Alexander Crichton states, that previous to the introduction of vaccination, every seventh child born in Russia died of small-pox.

The period was now approaching when it was to be discovered that a disease derived from one of the domestic animals would exert a powerful influence on the human frame. From the preceding enquiry, it appears certain that a fatal pestilential eruptive disease, common to man and the lower animals, has been known from the earliest period of authentic history, and that a somewhat similar if not perfectly identical disease yet exists in various regions of the earth, which disease is often fatal, but is subject to various modifications in respect to virulence, and is susceptible of further by artificial communication. It is thus understood how sheep, horses, and other animals may be subject to the disease, as well as cows or oxen; and that matter derived from the horse in the grease, the form of the disease in that animal, has produced the true vaccine disorder. The existence of the variolæ vaccinæ in the dairies of England not seeming to have been of long duration, Dr. Baron thinks there is good ground for believing that the disease, as originally noticed by Dr. Jenner, was but the endemic remains of the more extensive or epizöotic disease described by Layard.

"This opinion is strengthened by the following well authenticated facts. Soon after the publication of Dr. Jenner's discovery it was found that cow-pox existed in several counties of England—in Devon, Dorset, and Somerset, in Hampshire, Buckinghamshire, Middlesex, Wiltshire, Staffordshire, Norfolk, &c.; in all, eighteen counties. Accounts were also received of the existence of the variolæ vaccinæ among the cattle of Lombardy, Holstein and other regions where the pestilential eruptive disease, denominated *pestis bovina,* had previously raged, as recorded by Ramazzini and other medical writers. At a later period it was also found in Persia, both in cows and sheep, as appears in a letter from Mr. Bruce, English resident in Bushire, (11th vol. of the Edinburgh Medical and Surgical Journal, p. 270.) It was discovered also by Don F. X. Balmis among the cows of the valley of Atlixico near the city of Puebla de los Angeles; in the neighbourbood of Valladolid de Mechöacan, where the Adjutant Antonio Gutierrez found it; in the district of Calabozo in the province of Caraccas, by Don Carlos de Pozo, physician of the residence, and by Humboldt in the Peruvian Andes.

"In the Report of the Central Committee of Vaccination in Paris for the year 1821-22, it is stated that the disease has likewise been found in the vicinity of Clairveaux, by M. James Dubry; and to this list Ireland ought to be added on the authority of Dr. Barry, of Cork. From these facts it may very fairly be inferred that the variolæ will hereafter be found among cows in other parts of the world." (Vol. i. p. 239.)

A disease so widely observed cannot, Dr. Baron concludes, depend on mere local circumstances: and he looks upon its prevalence in every clime as another circumstance supporting the view of its affinity with small-pox. We do not admit the legitimacy of this last inference. Dr. Jenner, however, always maintained this view, and considered that in employing vaccine lymph he was only affecting the constitution with a mild instead of a virulent and contagious form of the disease; a view which has often been overlooked by disputants, with the effect of creating much useless discussion.

In a subsequent part of his first volume, Dr. Baron is enabled to allude to the recent prevalence of the variolæ vaccinæ in a malignant and fatal form among cattle. The report is given by an intelligent veterinary surgeon (Mr. Tiley) well acquainted with the disease. He was sent for, in 1825, to see a cow which was very ill, and found the cow's disorder to be aggravated cow-pox. On the teats were several blackish scabs, and the whole skin was one continued disease, "not of vesicles or scabs, but a discharge similar to that produced by a blister." There was violent fever, and the cow died. Between forty and fifty cows became similarly affected, but none died except the first. It was supposed that the infection was conveyed to the first cow by a servant of a neighbouring farmer, who was asked to milk it, and who daily dressed a greasy-heeled horse. All the servants who milked the affected cows had sore fingers, with one exception. In the second volume Dr. Baron adds an account of the epizöotic, from the report of Mr. M'Pherson, which prevailed in the northern parts of Bengal at a period (1832) subsequent to the publication of the first part of his work. It prevailed among cows, very extensively and severely; and Mr. M'Pherson procured matter from the cows so affected, and found it productive of the true vaccine disease, the protecting power of which was afterwards fully tested. The cows were affected with pustules all over the body.

It is pretty generally known, that soon after the publication of Dr. Jenner's first work, some of the first trials of vaccination were made at the Small-pox Hospital by Dr. Woodville; and the vaccine and variolous virus becoming commingled, an eruptive disease was produced, which, although milder than ordinary small-pox, was very different from "the benign solitary pustule" which characterizes the variolæ vaccinæ. The evil was unfortunately propagated all over Europe; but a fact of great value was, according to Dr. Baron, at least one of the results of this blunder; for it was proved "that even this matter, after repeated re-inoculations, lost much of its virulence; ceased altogether to produce eruptions; and at length became almost assimilated to the true vaccine character." (Vol. i. p. 245.) Two facts are here put in apposition by Dr. Baron: one, that the cow-pox sometimes affects the milkers with a severe disease; the other, that small-pox, after repeated inoculations, approximates in its nature to the mildness of cow-pox; the number of pustules gradually diminishing until only *one* is seen, and that at the place of insertion, efficiently protecting the constitution from subsequent small-pox, although running its course without constitutional disturbance. Had the result followed the inoculation with pure small-pox virus, the support thence afforded to Dr. Baron's and Dr. Jenner's views of the identity of the two diseases would have been vastly greater: but, in the case referred to, it was not true small-pox that was inoculated. In fact, the very circumstance of the vaccine character prevailing ultimately in this case, may be held as an argument against the identity of the diseases: for, if this identity existed, there seems reason for expecting that the results of this mixed infection should, in all future inoculations, exhibit some of the peculiar characters of each disease; there appearing no sufficient cause for the destruction of either: whereas, the contagions being considered essentially different, it might be supposed that they could not continue mingled without the character of one becoming predominant.

Those interested in this enquiry (and what medical man is not?) should by all means carefully peruse Dr. Baron's Eighth Chapter, which treats of the Difference between Variolæ and Variolæ Vaccinæ, and of Varioloid Diseases. It contains numerous facts, to which it would, as Dr. Baron says, have been easy to add many more, tending to show that if vaccination were steadily and carefully practised both the small-pox and the varioloid eruptions would disappear. The statements made of the mortality of small-pox may, indeed, now be usefully recalled to mind. Of all who were born, one in fourteen died of small-pox; and this even after the practice of inoculation was introduced. Of those affected by natural small-pox, one in five or six died; but of those who had been inoculated only one in fifty. "These conclusions were drawn by Dr. Jurin from an examination of the London bills of mortality for a period of forty-two years."

The other evil consequences of the disorder were many and great; but are too well known to require enumeration. The same may be said of the contrasted benefits of introducing a mild and non-contagious disease by vaccination ; but if any of our readers should entertain any doubts on this subject we would earnestly recommend him to refer to the evidence from all parts of the world contained in Dr. Baron's pages. The facts observed by Dr. Thomson in Edinburgh, in 1818-19, and by Mr. Crosse at Norwich, in 1819, are mentioned in this part of Dr. Baron's work. Their value is well known to the profession. Dr. Jenner had long before ascertained some of the points confirmed by these epidemics: he had shown that previous infection could not infallibly prevent susceptibility to the variolous poison: he had proved that by inoculating a person with small-pox matter who had been previously vaccinated, a local vesication might be produced from which virus could be obtained capable of producing a mild but efficacious form of small-pox; and he had maintained that the varieties in the vaccine pustule comprehended "every gradation in the state of the pustule, from that slight deviation from perfection which is quite immaterial, up to that point which affords no security at all." He subsequently pointed out that fluid taken from a spurious vaccine pustule can propagate its like; and that fluid taken from a genuine pustule when too far advanced may produce varieties which may be perpetuated, a circumstance which he did not in his first vaccinations suspect, and which other inoculators continued to neglect for some time. These truths have been too often forgotten.

We conceive it to be unnecessary in the present day, either in vindication of Dr. Jenner, or for any other purpose, to dwell on the errors and misconduct of Dr. Woodville and Dr. G. Pearson in relation to vaccination. The desire to usurp the merit belonging to another was in these cases singularly blended with a misapprehension of the doctrines and practice of him on whom they would have inflicted injustice and injury. It is melancholy to reflect on such conduct, too often seen, indeed, to arise from an ill-regulated wish for fame, unaccompanied with a sincere love of truth. Zealous vaccinators in this and other countries fell sometimes into the same mistakes as these physicians, and inoculated with small-pox virus or mingled virus, with unfortunate effects; but they did not exhibit the unhappy passions to which the above-mentioned vaccinators were too evidently a prey. Nor can we omit to mention that the late Lord Egremont, on the occurrence of some disastrous consequences in the neighbourhood of Petworth, the result of *contaminated* matter supplied by Dr. G. Pearson, applied his honest and excellent understanding with success to investigate the real cause of these supposed effects of cow-pox matter: he frankly communicated to Jenner the supposed effects (variolous-like eruptions) which had appeared, received Jenner's clear explanations, and with them a supply of real cow-pox virus, with which numerous vaccinations were made, with the happiest products. It affords us sincere pleasure to be able to say, that although Drs. Woodville, G. Pearson, and Moseley, distinguished themselves by very different conduct, there were not wanting many medical men who approached the subject candidly and calmly, and soon and fearlessly avowed their perfect confidence in the protection afforded by the genuine cow-pox. Among these Mr. Ring deserves honorable mention. Staggered by the effects of Dr. G. Pearson's distributions, he industriously enquired into their causes, and he was mainly instrumental in procuring a public testimonial in favour of vaccination, to which were appended, among others, the eminent names of Baillie, Cline, Cooper, and Abernethy.

If our design in this article were to notice every step in the history of vaccination, it would afford some interest to the reader, and certainly some instruction to detail the circumstances attending its introduction into America. The zeal and courage of Dr. Waterhouse, and the enlightened candour with which he met those reputed cases of severe vaccine disease which there, as elsewhere, were the product of carelessness or mistake, might be contemplated with unmixed pleasure. The patronage afforded to it by President Jefferson, and in France by Lucien Bonaparte and the scientific members of the Institute, and in Spain by the unusual energy of the government of that country, must have brought gratifying reflections to the mind of Jenner, and have consoled him under the very different treatment which he so often experienced in our own country. Mr. Jefferson devoted his attention (in 1802) to establishing the "*point of time*, counting from vaccination" (these are his own words), "when the matter is genuine in all cases;" and he decided, and Dr. Waterhouse confirmed his decision, that it was *eight times twenty-four hours;* which went forth, Dr. Waterhouse said, "like an edict." It was indeed an edict founded on intelligent observation. But Jenner was not without the active and useful support of many liberal-minded professional men in England: we have spoken of Mr. Ring and others, and the name of Mr. Dunning, of Plymouth-Dock, deserves to be especially recorded. By the judicious arrangements of Dr. Marshall and Dr. John Walker, vaccination was extensively introduced into the army and navy in Egypt, and also in Gibraltar, Minorca, Malta, and Sicily. In 1800, Sir Gilbert Blane, sanctioned by the late Earl Spencer, then First Lord of the Admiralty, effected the general introduction of the practice into the navy; a plan in which Dr. Trotter warmly co-operated. Dr. De Carro, of Vienna, whose name is so honorably known in connexion with vaccination, was the instrument of diffusing the blessings of the practice over the East; and it was greatly promoted at Constantinople and throughout the Archipelago by the exertions of Lord Elgin, (in 1802.) In Ceylon, in consequence of measures adopted by Governor North and Dr. Christie, vaccination became so general as very strongly to support the belief that if it were practised with equal industry everywhere, variolous contagion might be completely controlled. It was to Ceylon and to Sweden that Dr. Jenner used to refer when wishing to show what his discovery might accomplish.

In 1801, Jenner published his account of the Origin of the Vaccine Inoculation: a copy was sent to the Institute of France, and in the note which acknowledges it, signed by Coulomb, *Cuvier*, and Delambre, we think we detect the enlightened style of the illustrious naturalist: "to gather new truths from whatever quarter they may come, to spread them when they are useful, this is its first duty." These were surely the great views which Cuvier entertained of the duty of the institute.

When Jenner's discovery had been communicated to nearly every part of the globe, and honours had flowed in upon him from the princes and philosophers of several countries, the parliament of England took into grateful consideration the sacrifices made by him in the prosecution of his enquiries, and the labours of making them known wherever knowledge of any kind could penetrate. A previous contribution of a piece of plate was made to him in his own county of Gloucester, although Lord Berkeley, who zealously promoted it, found much supineness among the country gentlemen. It was necessary to approach parliament by a petition, which was referred to a committee, to whom evidence of the value of vaccination was given by some of the most eminent physicians and surgeons of the kingdom, and by distinguished unprofessional persons; including his late majesty, then Duke of Clarence. Dr. G. Pearson again appeared on the stage, as a witness, endeavouring to show that vaccine inoculation had been practised by others before Dr. Jenner; and that Dr. Jenner, although he promulgated the practice, knew very little about the matter, and that the experiments and observations of Dr. Woodville and himself were necessary to correct his mistakes. This, and a little more evidence to the same purport, had little effect upon the committee; except perhaps to create some misapprehension concerning the full extent of Jenner's merits. Dr. Baron sets the question in a fair light, when speaking of their report.

"The committee, while referring to the pretensions advanced in opposition to Dr. Jenner's claims, took, on the whole, a just view of these pretensions; but they were not equally correct in estimating the real nature of his merits. They truly observed that in various parts of England an opinion was current, among the common people employed in dairies, that the cow-pox was a preventive of the small-pox. 'It appears,' they add, 'not improbable that in some very rare instances this knowledge was carried one step further, and that the cow-pox was communicated either by handling the teat or by inoculation from the animal, for the purpose, and with the intention of securing against the danger of small-pox; but the practice of which Dr. Jenner asserts himself to be the original inventor is the inoculation from one human being to another, &c.' With all deference to the committee, it must be remarked that this forms but a very small part of his claims to public gratitude and remuneration. His chief merits are, as has been already proved, of a very different description. They consist in the patient, laborious, and original investigations which enabled him to extract correct and scientific information from the most unpromising materials; to divest popular tradition of all its obscurity and uncertainty; and to elicit from vague and contradictory rumours the most accurate and valuable truths. It was not till all this was achieved that he ventured to perform his first inoculation: and, had it not been so, this most admirable and interesting experiment had been as valueless and inconsequential as the *alleged* vaccinations of Mrs. Rendall and Farmer Jesty." (Vol. i. p. 503.)

The parliament concluded by voting Dr. Jenner 10,000*l.*; a sum which in the estimation of all the respectable medical authorities of the time ought to have been doubled. But the government had at that time little to spare for discoveries calculated to preserve human life. The grant, inadequate as it assuredly was to Jenner's services, was very tardily paid. Two years after it was voted, he had not received a farthing of it. When at length paid, the fees of office, and other charges, took nearly one-tenth from the amount. But the English parliament did not forget Jenner. In 1806, after another and most complete enquiry, conducted by the College of Physicians, the further sum of 20,000*l.* was voted to him; accompanied with such expressions of esteem from the leading men of the time as were not less valuable than the grant itself. Dr. Pearson alone thought the first sum of 10,000*l.* too great; and renewed his opposition to Jenner in an Examination of the Report of the Committee. To this, as to Pearson's previous onsets, Dr. Jenner declined to reply, trusting to the goodness of his cause, to truth, and to the justice which is now done to his memory. We sincerely wish that all those who lay claim to the honour of discoveries would imitate, among his other virtues, this part of Dr. Jenner's conduct. Perhaps it is only those who are calmly confident of the truth of what they maintain who can do this: there is a restless search for novelty that may be felt by many who want the support of the belief that truth is great and will prevail.

From numerous medical societies Dr. Jenner received votes of thanks and congratulation conveyed in the warmest and most respectful language. His views were discussed in the Physical Society held at Guy's Hospital, on four successive nights. Jenner himself attended, and was greeted "with universal and rapturous applause." These and other honours from the profession were deeply gratifying to him. Whatever faults may be laid to the charge of the medical profession, the general body has never been found insensible to the merit of a really great man. The tendency of our professional studies is to generate habitual caution, but this is a habit very distinct from any unwillingness to acknowledge genuine merit and undeniable services rendered to science. We have already had occasion to observe that many of the clergy exerted themselves zealously to diffuse a knowledge of Jenner's discovery. The first English clergyman who addressed his parishioners on the subject from the pulpit was the late Rev. Dr. Booker, of Dudley; a gentleman of some literary pretensions, and much vivacity, and whose lot was the somewhat singular one of living to record in his old age the virtues of *three* beloved wives on one tombstone.

One very extraordinary claim to the priority of discovery in the instance of vaccination we cannot pass over in silence. M. Husson, who, in 1803, emphatically expressed his opinion in favour of Dr. Jenner, wrote to him in highly complimentary phrase, did him justice in a published work, and even avowed his intention of coming to England merely to be able to say that he had seen him,—this same M. Husson, in 1821, in an article in the *Dictionnaire des Sciences Médicales*, recounts among the literary and scientific thefts of England, that of the discovery of vaccination, of which he asserts the first idea was suggested by a Frenchman; and he declares that a Frenchman (M. Ribaut) conceived the idea of communicating the cow-pox to man in 1781, and mentioned it before an English physician, who communicated it to Dr. Jenner. Independently of other disproofs, it seems scarcely necessary to say more than that Jenner's enquiry commenced five years before the date assigned to this communication. M. Husson had read the Report of the Committee of the House of Commons, and quoted it freely: he must therein have seen that Dr. Jenner communicated the result of his past enquiries to Mr. E. Gardner, in 1780. It does certainly appear, however, that M. Ribaut, the protestant minister at Montpelier, had imagined from his observation of a disease resembling small-pox in sheep, and *perhaps* from what he had heard of it as it appeared in cows, that it would possibly be advantageous to inoculate man from the cow; but there is not a shadow of proof that his opinion was ever, in any way, made known to Jenner; and not the smallest reason for supposing it possible that a man of Jenner's candid noble nature would have concealed the source of such a suggestion, even if, instead of being subsequent, it had been prior to that of his own mind. But to such vexations may an honest man be exposed. Jenner had to bear many such, and among them one or two attempts, which we feel assured were fraudulent, to trace his discovery to passages in ancient Sanscrit writings. Another trial to him must have been the natural death of the Royal Jennerian Institution; which, although originating (in 1803) in the purest motives, among the most honorable men of the profession, and patronized by persons of the highest rank, and for a time most active and most useful, perished (in 1808) by some irregularities in the proceedings of some of its officers; more especially of Dr. Walker, whose instructions respecting vaccination deviated so much from those of Jenner as to compel him to bring charges against Dr. Walker, who was, however, retained in office by a majority of three, notwithstanding full proof of the charges. This was effected by one of those disgraceful acts which now and then occur in the management of public institutions; several votes (twenty) being created by money for the occasion. From that time the society, if it can be said to have had any existence, has been in no way, we believe, conducted on the principles of the great name it very improperly retained. By such events was doubtless somewhat counteracted the pleasure which Jenner must now have derived from the consciousness of his widely and rapidly extending fame; for in six years from its first promulgation the discovery of vaccine inoculation was known in every region of the world: "proceeding eastward and westward," says Dr. Baron, "it traversed the circumference of the globe."

In 1804 arose the belief, now by many entertained, and which seems to us far from groundless, that the cow-pox afforded only a temporary security. This belief, however, is considered by Dr. Baron to rest on no solid foundation. The cases brought forward by Goldson to support this objection were, he says, from the first, perceived by Jenner to be cases in which the cow-pox had never properly taken place. Mr. Dunning is honorably mentioned as setting this question in a clear point of view. Like Jenner, he had become acquainted with the benefits conferred by vaccination in consequence of his study of small-pox; and it ought ever to be borne in mind, says Dr. Baron, that Jenner's knowledge respecting small-pox was the medium of his discovery of the laws which govern the variolæ vaccinæ. It had been shown that, in the inoculation for small-pox, the mitigation of the symptoms depended much on the number of pustules produced; and that even *one* pustule could afford protection. "To moderate the eruption and to subdue the fever was the object of all the enlightened physicians who treated small-pox. Inoculation most materially aided these intentions; vaccination carried them into full effect." (Vol. ii. p. 18.)

It must, doubtless, be trying to the patience of those friends of Dr. Jenner, who have carefully kept his principles in view, to see them lost sight of and neglected, and out of this neglect questions and doubts again and again arising, in defiance of former refutations. Thirty-four years ago, some of the points which have been discussed within the last few years were, Dr. Baron observes, fully explained.

"It was then clearly ascertained, that there were deviations from the usual course of small-pox, which were quite as common and infinitely more disastrous than those which took place in vaccination. These deviations regarded two apparently different states of the constitution. In the one, the susceptibility of small-pox was not taken away by previous infection; while, on the other hand, some constitutions seem to be unsusceptible of small-pox infection altogether. It was found, that similar occurrences took place in the practice of vaccination; but as the security which the latter afforded was more likely to be interfered with by slight causes than the former, it became absolutely necessary that great care should be shown in watching the progress and character of the pustule. Dr. Jenner had from the beginning felt the propriety of this watchfulness; and had distinctly announced, that it was possible to propagate an affection by inoculation conveying different degrees of security, according as that affection approached to, or receded from, the full and perfect standard. He also clearly stated, that the course of the vaccine pustule might be so modified as to deprive it of its efficacy; that inoculation from such a source might communicate an inefficient protection, and that all who were thus vaccinated were more or less liable to subsequent small-pox." (Vol. ii. p. 20.)

We fear Dr. Jenner's directions for avoiding these occurrences have often been disregarded; and the character of the pustule, and the time and quality of the lymph, and the integrity of at least one pustule, and the general state of the skin, too much overlooked. Many obstacles have everywhere presented themselves to the systematic observation of the progress of the vesicle; and hence, we feel strongly assured, the cases of partial or inefficient protection have been very numerous, and the cases of complete protection proportionably diminished. The opinion expressed by Mr. Dunning, in 1806, may be probably correct, that the constitution loses its susceptibility to small-pox contagion in proportion to the perfection of the vaccine vesicle; leaving the constitution incapable of producing small-pox in its ordinary form in cases where the vesicle has been perfect, or modifying its subsequent appearance according to its approach to that perfection.

Two facts are mentioned in this part of Dr. Baron's work, which have so important a bearing on the question of second attacks of small-pox that we cannot resist inserting them in this place. One is the fact mentioned in a letter of Dr. Jenner to Mr. Dunning, that the surgeon of the South Gloucestershire militia told him he was so susceptible of the contagion of small-pox that he never attended a patient in that disease without catching it. The other is mentioned by Dr. Baron himself, in a note, (vol. ii. p. 26,) and relates to the child of a cousin of Dr. B.'s, who was vaccinated in India, apparently with success, and subsequently in England, again receiving the infection; and who was afterwards inoculated for small-pox, and took the disease, and at a still later period was exposed to its influence, and had it again. "Could a stronger illustration," remarks Dr. Baron, "be adduced of the doctrine laid down in these volumes touching the identity of the diseases in question, with relation to their protecting power?" We could ourselves give several cases of equal authenticity and weight; but we see in them no proof of the identity of the two diseases, although a striking similarity in their protective powers.

In this part of the work, also, Dr. Baron inserts a few passages from one of Dr. Jenner's journals, containing remarks which he considers subsequent investigations admirably to have confirmed the sagacity of. The following are the passages:

"The origin of small-pox is the same as that of cow-pox; and, as the *latter* was probably coeval with the brute creation, the former was only a variety springing from it.

"There are certainly more forms than one, (without considering the common variation between the confluent and distinct,) in which the small-pox appears in what is called the natural way.

"It will be enquired (if the foregoing reasoning be *à priori* correct,) in what way can the action of cow-pox, (or the equine pock,) in preventing subsequent small-pox, be reconcilable with the established laws of the animal economy? My reply is, for the reasons which I have stated on the basis of facts, that they were not *bonâ fide dissimilar* in their nature; but, on the contrary, *identical.* On this ground I gave my first book the title of 'An Inquiry into the causes and effects of the *Variolæ Vaccinæ,*' —a circumstance which has since been regarded by many as the happy foresight of a connexion which was destined, by future evidence, to become more warranted." (Vol. ii. p. 30.)

Passing over many interesting incidents in the history of vaccination, and in the personal history of Dr. Jenner, although related in so touching and beautiful a manner that to pass the latter over is not easy, we find, in the eighth chapter of the second volume, a candid summary of the facts relating to vaccination. In this chapter Dr. Baron considers the important questions, 1, of the cow-pox retaining the distinctive marks which characterized it when first discovered; and, 2, of its having lost in any degree its original prophylactic powers. Dr. Baron fully believes that the cow-pox is now what it was at the beginning. There are instances in which it has passed from one human subject to another for more than thirty years, consequently through fifteen or sixteen hundred individuals, and yet in which no degeneration has taken place. (Vol. ii. p. 244.) He nevertheless thinks it proper to employ recent lymph from the cow, when it can be procured. Dr. Baron acknowledges that, in some cases, "small-pox has occurred after the most perfect vaccination;" but he thinks such cases do not exceed in number the cases of small-pox after small-pox. Our own experience and enquiries, we must say, lead to an opposite conclusion; and we think the general opinion of the profession coincides with ours.

It is unnecessary for us to dwell on the share vaccination has had in the improvement of the health of the community, and in the diminution of the mortality among infants. Upon these points, notwithstanding the erroneous conclusions of Dr. Watt, of Glasgow, medical men are pretty well satisfied. Its benefits in protecting infirm constitutions from a disease so often followed by the worst consequences, cannot be accurately calculated; but they must be very considerable. As regards the complete extirpation of small-pox by the practice of vaccination, Dr. Baron does not feel discouraged by the variolous epidemics which have of late years appeared in Lombardy, Denmark, France, and England. In all his professional life, he has only seen one *fatal* case of small-pox after vaccination: but surely he must be well aware that many such cases are on record: indeed, there are few practical *surgeons* who have not seen some such.

For the enormous proportion of successful revaccinations in the Danish army, (2175 in 3173,) he would account by the first vaccinations being imperfect; and he would apply the same remark to the revaccinations in Prussia. This is, to say the least, a summary way of getting over the difficulty. In our next Number, we hope to place this question in its true light, in an analysis of the recent elaborate work of Dr. Heim.

This brief account of some of the events connected with the important subject of vaccination might, we have conceived, be with some advantage placed before the reader. No one, we think, can go minutely through the details which we have but imperfectly condensed, without deriving from them a conviction of the stability of the greater number, if not of every one of the points which Dr. Baron is anxious to represent as firmly established. That Jenner was the first to think of protecting the human constitution from the small-pox by the artificial introduction of the cow-pox, is not a matter which any reasonable man is now inclined to dispute. That the disease in the cow, as observed by Jenner, was but the remains of a more severe and general affection of the skin and mucous membranes of the animal; and that such an affection, in one form or other, had been prevalent at different times, both in the cow and in other animals, from a remote period; and that one form of this affection was what is commonly called the *grease* in the horse, is also, we are of opinion, supported by strong evidence. That this affection, artificially or accidentally introduced into the human system, can produce an affection of different degrees of severity, but usually of singular mildness, and confined to a solitary pearl-like pustule, is sufficiently established; and that this affection, in proportion to its completeness or modifications, protects the constitution from subsequent small-pox, in a very great proportion of cases, and for a period of years at least, if not for life, we entirely believe. That its failure to do this has, in a vast proportion of cases, arisen from inattention to the character of the cow-pox affection in those from whom lymph has been taken, and to the period or age of the lymph; and to the condition of the skin and constitution of those into whose system it was introduced by inoculation, we entertain a very strong conviction. We feel no surprise at what we consider but so many proofs of the carelessness of early vaccinators; in which, among other negligences, it is notorious that very often not one vesicle was allowed to remain undisturbed. That in the great majority of cases in which pure lymph is employed, according to its degree, there is, as a result, a protecting quality imparted, but only more or less modified, we can scarcely doubt: and, when all these circumstances have obtained due attention, we are convinced that the cases in which the constitution has not been protected against small-pox will be found to have exceeded in number the cases of well-attested second attacks of small-pox, in a much smaller proportion than is now generally believed. It remains, we think, still to be proved that, where all Dr. Jenner's directions have been attended to, the vaccine lymph has undergone the smallest deterioration. Against making any or all of these points the subject of new and most accurate experiment and observation, no objection can, of course, be entertained: on the contrary, we think that, in the present state of the public mind, such experiments are necessary; but we cannot too strongly recommend to all vaccinators, and more especially to the vaccinating officers of institutions in large towns, to consider how responsible and important a duty they are engaged in; how mischievous their labours may be, if negligently performed; how full of benefits to the whole community, if conscientiously attended to. Neither Dr. Baron, nor Dr. Jenner himself, if he were living, would object, we feel assured, to anything by which the truth might be made more clear, the public mind confirmed, and the trust in vaccination rendered so secure that the practice might become universally successful.

If such should be the result, the yet-disputed question of the actual identity of the cow-pox and small-pox becomes of minor importance. There is an evident and strong analogy between the two affections; and, generally, those who have had either are unsusceptible of the other, and those who are by natural constitution unsusceptible of one are equally unsusceptible of the other: but, as the artificial communication of the cow-pox from the lower animals to man, or from one human subject to another, has never produced a general eruption, or true small-pox, (although repeated small-pox inoculations have reduced the eruption to *one* pustule, and although Mr. M'Pherson's inoculations of the human subject from cows covered with a pustular disease produced true vaccine disease, and furnished protective virus;) and as the inoculation of the cow with small-pox matter does not appear hitherto to have been satisfactorily proved to produce cow-pox; the evidence of the identity of cow-pox and small-pox must be considered as yet incomplete, without any prejudice to vaccination. The good to be expected from it, making all possible deductions, is, the *almost complete* extirpation of small-pox; a disease which, if now let loose upon the increased population of this country, would sweep into the grave more than 50,000 persons in every year, and leave many times that number in a state of debility which would predispose them to fall victims to other diseases, probably after becoming the parents of debilitated children; for no single disease ever contributed so largely and so directly to the deterioration and destruction of human beings as small-pox. Even admitting that the first cause of small-pox may be some unknown atmospherical quality, occasionally present, its presence would be rendered nearly innocuous from the want of a number of unprotected constitutions. Cases of small-pox would still occur,—some in those previously vaccinated, some in those who had previously had small-pox; but the disease would not spread; and, unless mankind, relieved from fear, should also become utterly devoid of caution, no devastating small-pox epidemic would be heard of more.

It would be difficult for us to express the gratification with which we have read the account of Dr. Jenner's Life and Character. His biographer sustains throughout a high and becoming tone, worthy of his subject and of himself. There is no base flattery of the living, nor any unmeasured condemnation of the dead; but everything is spoken of in a manner at once calm and courageous; and the opinions given are evidently guided by the strictest love of justice. We admire Dr. Baron's affectionate but not injudicious zeal in defence of Jenner's reputation, and accept with gratitude the details into which he has not thought it unworthy of biography to enter, of the personal appearance, and even the dress, of the great discoverer of vaccination. Among the many examples of spirited and admirable narrative, in various parts of the work, we might quote the account given of Dr. Jenner's interview with the Duchess of Oldenburg and the Emperor Alexander: and in the description of Jenner's love of natural scenery, that of Barrow Hill, one of his most favorite spots, and that of the Chantry, his residence at Berkeley, are depicted with a force and beauty which every reader of sensibility will appreciate. We can readily conceive that it must have been impossible to know Jenner so intimately as it was Dr. Baron's privilege to do, and not to be charmed with his character. In going over the details compressed into the preceding pages, we have again and again been struck with admiration of his many excellent qualities: of his candour in communicating his reflections, in every stage, to all mankind; of his inexhaustible patience in explaining his views to all sorts of writers, in a correspondence, as he used to say, with the world; of his personal activity as a vaccinator; of his equanimity under all sorts of claims made to his prejudice; of his forgiveness of injuries in the case of Dr. Woodville and others; and of the acute sensibility and dignity of mind which were in him so exquisitely blended with true humility, and a philosophical calmness that not the most insolent abuse could long disturb. In these days it is scarcely credible that the opponents of a discovery, of which the probable effect was to extirpate a scourging malady, appealed to the violent prejudices of the vulgar, by means of prints representing the human visage transformed into the likeness of that of a cow. With all our knowledge of the unprincipled character of the public press, we read with surprise that the "Cow-pox Chronicle" was a publication devoted to assailing the vaccinators with unlimited periodical ribaldry; and we can scarcely believe that the public were so brutally stupid less than half a century ago, that, after a public discussion in what unworthily bore the name of the British Forum, it should be with solemn ignorance decreed that gas-lights and cow-pox were mere instances of popular credulity. Still less credible does it appear that, when his grant was under discussion in parliament, one of the arguments used against it was, that Dr. Jenner might have made a fortune by keeping the nature of his discovery secret. Heavier injustice was often done to Jenner. In the establishment of the National Vaccine Board, in 1809, he was nominally constituted director; but he was compelled to resign by finding he had no influence. Although his name was a safe passport to the traveller over Europe, and Napoleon released prisoners on his intercession, he was unable, at any time, to get any appointment for any of his relatives from our own government, or any preferment in the church for his nephew. The College of Physicians, ever consistent with its principles, overruled the generous desire of Baillie, and other ornaments of their body, and withheld the fellowship from him. By acts like these the college has incurred the mortification of seeing its honours refused by those to whose names its honours could add nothing. Neither these slights nor the wit of his enemies disturbed, although they might sometimes wound, the pure spirit of Jenner. He felt a holy reliance in the truth of his discovery, which he well knew could not long be concealed by any acts or efforts of man. To his livelier friend Mr. Ring, who had indignantly satirized the detractors from his fair reputation, he says, "Your satires against the anti-vaccinists are keen; but the keenest of all are those which you engrave *with the point of your lancet.*" Of his more serious sources of consolation Dr. Baron quotes many proofs, which could scarcely with propriety be transferred to our pages. One of them we find in the delightful *letters* appended to the second volume: writing to the Rev. Mr. Clinch, of Newfoundland, when packing up for Cheltenham, Jenner says, " Never aim, my friend, at being a public character, if you love domestic peace. But I will not repine: nay, I do not repine, but cheerfully submit, as I look upon myself as the instrument in the hands of that Power which never errs, of doing incalculable good to my fellow-creatures." These views are further alluded to in the following account, by Dr. Baron, of Jenner's general temper of mind.

"Though the general cast of his character exhibited a happy union of great solemnity and seriousness with extraordinary playfulness, amounting at times even to the height of mirth and jocularity, yet no one ever found these latter qualities misplaced, or obtruding themselves unseasonably. Almost all the great incidents of his life tended rather to suppress them, and to keep them in the shade. In the early part of this work it has been shown, when meditating on the grand results of his vaccine experiments, how devout were his feelings. Towards the close of his life many incidents argue the increasing power of that principle. He frequently expressed his regret that mankind were so little alive to the value of vaccination. Among the last words that he addressed to me, not many days before his fatal seizure, he used this remarkable expression: 'I am not surprised that men are not thankful to me; but I wonder that they are not grateful to God for the good which he has made me the instrument of conveying to my fellow-creatures.'" (Vol. ii. p. 295.)

After several serious interruptions of his health, Dr. Jenner had an alarming attack of faintness, giddiness, and insensibility, in August 1820; he being then seventy-four years of age. This attack left no paralysis, and, after a little needful repose of his faculties, he became gradually, and in the course of a few months, as much engaged as ever in his usual occupations. His astonishing industry still continued, and his habitual cheerfulness seldom deserted him. At length, after a busy day, he retired to rest as usual, on the 24th of January, 1823. The next morning he arose at his accustomed hour, and came down stairs to his library. Not making his appearance at the breakfast-table, his servant was sent to him, and found him lying on the floor, apoplectic. There was general insensibility; and life was extinguished early on the following morning: an easy, placid, and appropriate death for him whose studious hours had ever been devoted to effecting good ends. Such was the tranquil end of Black, who died in his chair in his laboratory, like one who had fallen, after life's labours, into a sweet sleep; and such the end of Petrarch, who was found leaning on a book in his library, resting peacefully amidst the memorials of elegant letters in which the happiest part of his life had been spent. Jenner, like Cuvier and other naturalists, had contemplated the approaches to this termination of human life. Seven years before his death, in one of his many admirable letters to Mr. Moore, he mentions some warning symptoms, and adds, "I am tolerably easy about it, as at my time of life I must expect to see and feel the preparation for the extinction of vitality."

Not many days before Dr. Jenner's fatal attack, he wrote this parting statement respecting vaccination on the back of a letter, constituting perhaps the last words recorded by him on the subject. We agree with Dr. Baron, that "nothing could be more solemn, whether we consider the time or the expression of this his final judgment." And with these words we conclude.

"My opinion of vaccination is precisely as it was when I first promulgated the discovery. It is not in the least strengthened by any event that has happened, for it could gain no strength; it is not in the least weakened, for, if the failures you speak of had not happened, the truth of my assertions respecting those coincidences which occasioned them would not have been made out." (p. 311.)

We recommend Dr. Baron's volumes, in the strongest terms, to the attention of all our readers,—young and old, medical and surgical.

